# Probing of *Actinobacillus pleuropneumoniae *ApxIIIA toxin-dependent cytotoxicity towards mammalian peripheral blood mononucleated cells

**DOI:** 10.1186/1756-0500-1-121

**Published:** 2008-12-01

**Authors:** Philippe GAC Vanden Bergh, Laurent LM Zecchinon, Thomas Fett, Daniel Desmecht

**Affiliations:** 1 Pathology Department, Faculty of Veterinary Medicine, University of Liege, Colonster Boulevard 20 B43, B-4000 Liege, Belgium

## Abstract

**Background:**

*Actinobacillus pleuropneumoniae*, the causative bacterial agent of porcine pleuropneumonia, produces Apx toxins which belong to RTX toxin family and are recognized as the major virulence factors. So far, their target receptor(s) has not been identified and the disease cytopathogenesis remains poorly understood. Production of an active Apx toxin and characterization of its toxic activity constitute the premises necessary to the description of its interaction with a potential receptor. From this point of view, we produced an active recombinant ApxIIIA toxin in order to characterize its toxicity on peripheral blood mononucleated cells (PBMCs) isolated from several species.

**Findings:**

Toxin preparation exercises a strong cytotoxic action on porcine PBMCs which is directly related to recombinant ApxIIIA since preincubation with polymyxin B does not modify the cytotoxicity rate while preincubation with a monospecific polyclonal antiserum directed against ApxIIIA does. The cell death process triggered by ApxIIIA is extremely fast, the maximum rate of toxicity being already reached after 20 minutes of incubation. Moreover, ApxIIIA cytotoxicity is species-specific because llama, human, dog, rat and mouse PBMCs are resistant. Interestingly, bovine and caprine PBMCs are slightly sensitive to ApxIIIA toxin too. Finally, ApxIIIA cytotoxicity is cell type-specific as porcine epithelial cells are resistant.

**Conclusion:**

We have produced an active recombinant ApxIIIA toxin and characterized its specific cytotoxicity on porcine PBMCs which will allow us to get new insights on porcine pleuropneumonia pathogenesis in the future.

## Background

*Actinobacillus pleuropneumoniae *is the bacterial causative agent of porcine pleuropneumonia, a frequent and highly infectious disease generating significant economic losses related to deficits in zootechnical profits and intensive use of antibiotics [1,2]. Bacterium virulence factors include exotoxins Apx («*A*. *pleuropneumoniae *RTX») -IA, -IIA, -IIIA and -IVA, lipopolysaccharides, polysaccharidic capsule, fimbriae, iron collecting systems, proteases, superoxide dismutase, etc [3]. Among these weapons, Apx toxins are recognized as major virulence factors. These exotoxins belong to RTX (Repeats in ToXin) proteins family. These latter molecules share the same structural characteristic which is a series of glycine- and aspartate-rich nonapeptide repeats which constitute the main calcium-binding sites of the protein [4]. If the *apxIVA *gene is not disrupted by an insertion element [5], all serotypes are able to synthesize ApxIVA (only *in vivo*) whose autocatalytic and cross-linking activities [6] make it different from other Apx toxins which are of the pore-forming toxin (PFT) type.

Some of the Apx toxic activities were already detected by precedent studies. It is accepted that: (i) ApxIA exerts a strong hemolytic activity and a strong cytotoxic activity, (ii) ApxIIA possesses a weak hemolytic activity and a moderate cytotoxic activity, and (iii) ApxIIIA does not display a haemolytic activity but a strong cytotoxic activity on porcine neutrophils and pulmonary alveolar macrophages (PAM) [3,7-10]. Contrary to LtxA (*Aggegatibacter actinomycetemcomitans*), LktA (*Mannheimia haemolytica*), HlyA (*Escherichia coli*) and CyaA (*Bordetella pertussis*) RTX toxins for which it was shown that they acted through *β*_2_-integrin receptors to induce a cytotoxic effect on leukocytes [11-18], the target receptor of PFT Apx toxins has not been identified yet and cytopathogenesis of associated disease remains poorly understood. In this perspective, active Apx toxin production and characterization of its toxic action constitute premises necessary to the description of its interaction with a potential receptor.

## Methods

### Preparation of rApxIIIA toxin

Plasmid pJFF1003, containing ApxIIIA gene, was kindly provided from P. Kuhnert and J. Frey (Institute of Veterinary Bacteriology, University of Bern, Switzerland). This plasmid contains, inserted in the vector pET14b, the *apxIIICABD *operon controlled by a strong constitutive endogenous promoter [19]. Transformed *E. coli *Rosetta™ (Novagen, Belgium) were seeded on Luria-Bertani (LB) agar plates with ampicillin (50 μg/ml) and incubated overnight at 37°C. Several clones were then cultivated, each one in 200 ml LB broth with ampicillin (50 μg/ml) and the Complete^® ^protease inhibitor cocktail (Roche, Belgium), one tablet for 50 ml culture with shaking (200 rpm) at 37°C until an optic density of 1.2 at 600 nm was reached. Next, the toxin was concentrated following Maier and collaborators protocol [19] and finally dissolved in sterile DPBS (1 ml for 200 ml of starting culture) (Lonza Biowhittaker, Belgium). The ImageJ 1.37c software [20] gave an estimation of ~100 μg/ml (~0.83 μM, ~5.10^14 ^toxins/ml) ApxIIIA (120 kDa) after electrophoresis on Coomassie blue-stained sodium dodecyl sulfate gels (Invitrogen, Belgium) by using 1 μg of BSA as standard.

### PBMCs recovery from several species

Fresh blood rescued from five pigs (*Sus scrofa domesticus*, Piétrain), three wild boars (*Sus scrofa scrofa*), two cows (*Bos Taurus*), two goats (*Capra hircus*), one llama (*Lama pacos*), a man, two dogs (*Canis familiaris*), ten mice (*Mus musculus*) and two rats (*Rattus norvegicus*) were taken in BD Vacutainer^® ^citrate tube (BD, Belgium). Peripheral blood mononucleated cells (PBMCs) were then extracted by the Accuspin™ System-Histopaque^® ^1077 (Sigma, Belgium) according to the manufacturer's protocol and resuspended to a density of 2.10^6 ^cells/ml in RPMI-1640 with 25 mM Hepes and L-glutamine (Lonza Biowhittaker, Belgium), supplemented with 10% [v/v] heat-inactivated fetal bovine serum (Lonza BioWhittaker, Belgium) along with amphotericin-B 250 μg/ml (Gibco, Belgium) and penicillin-streptomycin 10.000 U/ml (Lonza BioWhittaker, Belgium).

### Cell culture

PK15 cell line was purchased from the ATCC collection (CCL-33), maintained in EMEM (Lonza BioWhittaker, Belgium) supplemented with 10% [v/v] heat-inactivated fetal bovine serum (Lonza BioWhittaker, Belgium) along with amphotericin-B 250 μg/ml (Gibco, Belgium) and penicillin-streptomycin 10.000 U/ml (Lonza BioWhittaker, Belgium), at 37°C in a humidified 5% CO_2 _incubator and isolated from the flask after treatment with the Cell Dissociation Solution Non Enzymatic (Sigma, Belgium).

### Surface labeling of porcine leukocytes and flow cytometry analysis

Localization of the porcine PBMCs on the SSC (Side Scatter)/FSC (Forward Scatter) dot plot was accomplished by a PoLFA-1 cell-surface labeling. First, 10^5 ^cells were washed three times with 1 ml DPBS, 1% BSA, by a centrifugation of 5 minutes at 200 g. Next, cellular surface was blocked during 20 minutes on ice with 1 ml DPBS, 1% BSA, and then primary antibody (mouse anti-porcine CD18, MCA1972, Abd Serotec, Belgium) was added at 1/1,000 dilution and incubated for 20 minutes on ice. Cells were washed again three times with DPBS, 1% BSA, and the secondary antibody (AlexaFluor^® ^488 goat anti-mouse IgG, Molecular Probes, USA) was added at 1/1,000 dilution for 20 minutes on ice. Finally, leukocytes were washed three times with DPBS, resuspended into 500 μl of this buffer and analyzed for AlexaFluor^® ^488 fluorescence on a BD FACSCanto™ flow cytometry system using BD FACSDiva software (Becton Dickinson, Belgium). Labeling with an isotype-matched non pertinent murine mAb was used as negative control.

### Cytotoxicity analysis by flow cytometry

In order to undertake the cytotoxic assays, 5 μg rApxIIIA (50 μl stock solution) were added to 10^5 ^PBMCs in 50 μl of RPMI-1640 or 10^5 ^PK15 cells in 50 μl of EMEM. The positive and negative controls of cell death were obtained by adding respectively 50 μl paraformaldehyde 10% (Sigma, Belgium) and 50 μl RPMI-1640 or EMEM (mock-exposed) to 10^5 ^cells into 50 μl medium. Development of cell death was stopped by the addition of 1 ml of ice-cold DPBS and pellets were then suspended into 495 μl DPBS plus 5 μl propidium iodide (PI) (250 μg/ml) (Invitrogen, Belgium), after a centrifugation of 5 minutes at 200 g. Finally, the cells susceptibility to rApxIIIA was assayed by measuring PI fluorescence on the BD FACSCanto™ flow cytometry system. Each experiment was made in triplicate.

## Findings

### Flow cytometric probing of toxin-induced alterations of porcine PBMCs

**Figure 1:**
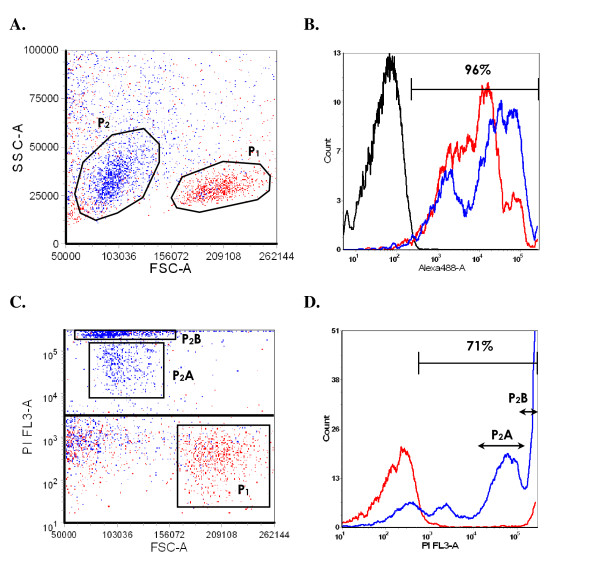
**A. Spreading of porcine PBMCs elements in SSC/FSC dot plot after rApxIIIA addition**. P_1 _(red) and P_2 _(blue) populations represent mock- (RPMI-1640) and rApxIIIA-exposed porcine PBMCs respectively. X-axis and Y-axis represent FSC and SSC values respectively. B. Surface expression of LFA-1 by mock- (P_1_, red) and rApxIIIA (P_2_, blue) exposed porcine PBMCs. Surface labeling was made with anti-PoCD18 mAb MCA1972. Labeling with an isotype-matched nonpertinent murine mAb was used as negative control (black). X-axis shows fluorescence intensity (Alexa 488) and the Y-axis represents cell count. The percentage of Alexa 488-positive cells is indicated within the panels. C. Spreading of porcine PBMCs elements in PI/FSC dot plot after rApxIIIA addition. P_1 _(red) and P_2 _(blue) populations represent mock- and rApxIIIA-exposed porcine PBMCs respectively. Two subpopulations can be observed for P_2_: the first shows an intermediate labeling (P_2_A) while the second (P_2_B) is very strongly tagged, corresponding to intermediate and final stages of cell death respectively. The bar represents the positivity threshold. X-axis and Y-axis represent FSC and PI-fluorescence values. D. Distribution of PI labeling among mock- (red) and rApxIIIA-exposed (blue) porcine PBMCs. The P_2_A and P_2_B subpopulations are readily detected. X-axis shows fluorescence intensity (PI) and the Y-axis represents cell count. Percentage of PI-positive cells is indicated within the panels.

Flow cytometric analysis of physical characteristics of porcine PBMCs, using SSC/FSC dot plot, revealed a principal cell population (P_1_) (Fig. 1A). Anti-porcine (Po) CD18 mAb bound near 100% of P_1 _cells (Fig. 1B), confirming that it corresponds to the porcine PBMCs population. P_1 _is characterized by FSC and SSC values which presumably correspond to porcine lymphocytes.

After a one hour-incubation with 5 μg rApxIIIA crude toxin, the SSC/FSC signature of P_1 _PBMCs is displaced towards a P_2 _population with reduced FSC values (Fig. 1A). Again, surface labeling with the anti-PoCD18 mAb showed that near 100% of P_2 _cells correspond to PBMCs (Fig. 1B). Exposition to rApxIIIA thus resulted in a reduction in size with no modification of granularity (SSC). This cellular morphologic change is compatible with the induction of a cell-death process by ApxIIIA [21].

Moreover, in the PI/FSC dot plot (Fig. 1C), it was observed that P_2 _is subdivided into a very strongly stained population (P_2_B) and another showing an intermediate labeling (P_2_A), presumably corresponding to intermediate stages of cell damage. The cell counts/PI histogram (Fig. 1D) revealed that approximately 70% of porcine PBMCs underwent a cell-death process.

### Kinetics of crude toxin preparation cytotoxicity on porcine PBMCs

**Figure 2:**
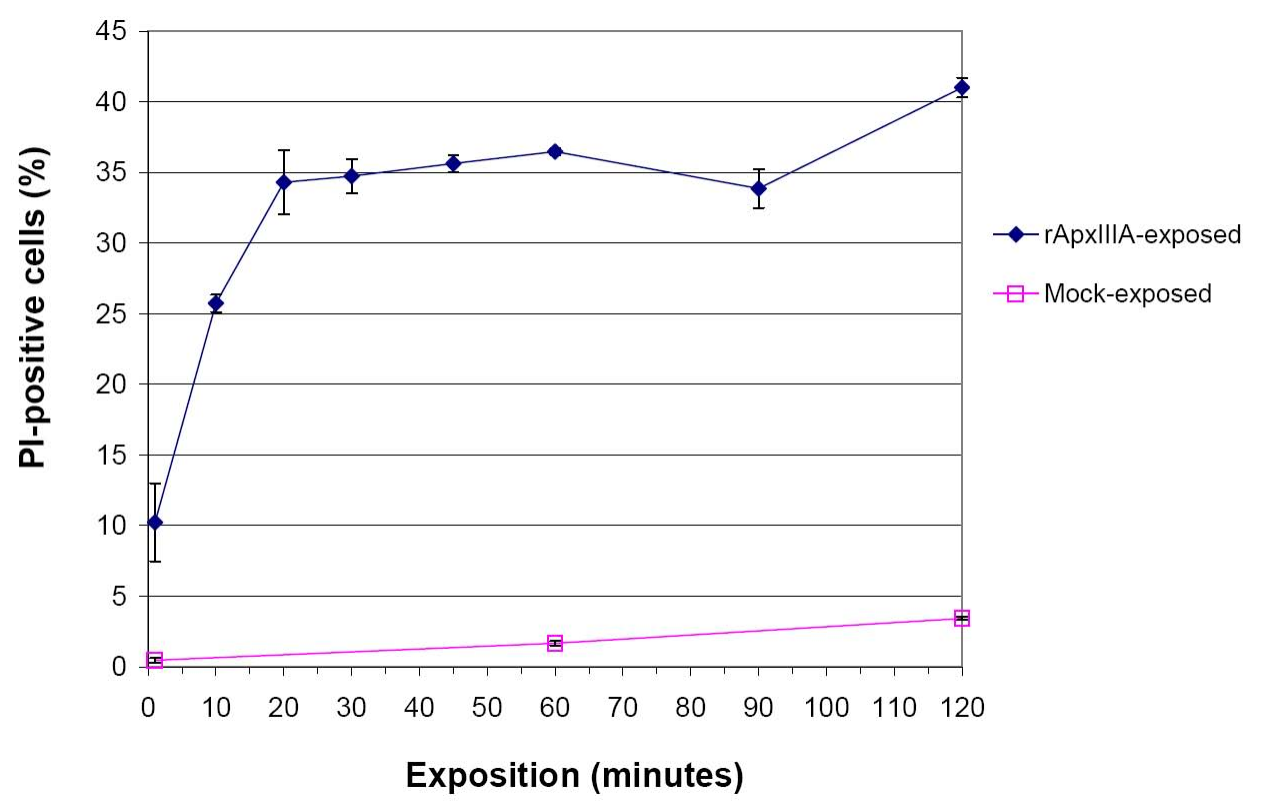
**Kinetics of porcine PBMCs death upon exposition to rApxIIIA crude toxin preparation**. Kinetics of the cytotoxic action was characterized by incubating porcine PBMCs with 0.31 μg rApxIIIA crude toxin preparation and stopping the exposure after 1, 10, 20, 30, 45, 60, 90 and 120 minutes. Horizontal axis, duration of exposition; vertical axis, fraction of PI-positive PBMCs. Basal cell death rate was measured from mock-exposed (RPMI-1640) PBMCs for comparison. Values are means ± SDs from three representative experiments.

Kinetics of the cytotoxic action was characterized by incubating 10^5 ^porcine PBMCs in 50 μl of RPMI-1640 with 0.3125 μg rApxIIIA crude toxin and stopping the exposure after 1, 10, 20, 30, 45, 60, 90 and 120 minutes. The results show that cell poisoning is an extremely rapid process. Indeed, one minute incubation is already sufficient to lead to a cytotoxicity rate of approximatively ten percents (Fig. 2). Moreover, a plateau of maximal cell death (~35%) is reached after 20 minutes of incubation (Fig. 2). This speed of action was also observed in a precedent study in which marked morphological changes occurred in pulmonary alveolar macrophages within ten minutes of exposure to ApxIIIA [22]. After this period, the toxicity rate seems to increase slightly, following the kinetics of negative controls (Fig. 2).

### Dose dependent-cytotoxic activity of crude toxin preparation on porcine PBMCs

**Figure 3:**
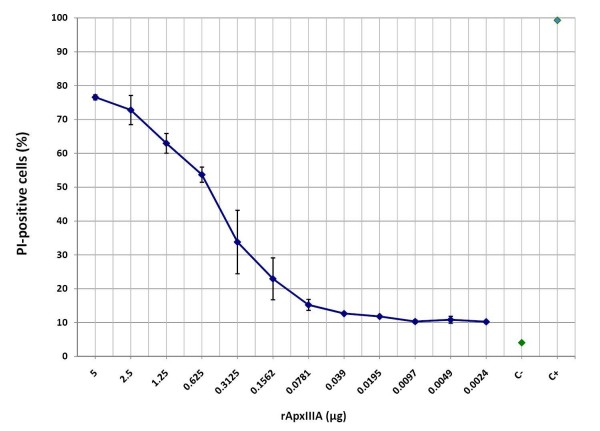
**Dose-dependent cytotoxicity of rApxIIIA on porcine PBMCs**. The fraction of PI-positive cells was measured one hour after exposition to serial dilutions of the crude toxin preparation. Values are means ± SDs from three representative experiments. C-, mock-exposed (RPMI-1640) porcine PBMCs incubated for one hour (negative control); C+, paraformaldehyde-exposed PBMCs (positive control).

As expected, porcine PBMCs were damaged in a concentration-dependent manner when exposed to different dilutions of rApxIIIA crude toxin. Maximum of toxicity rate (~80%) was obtained with the addition of 5 μg toxin (50 μl stock solution) to 50 μl RPMI-1640 with 10^6 ^porcine PBMCs (Fig. 3). Moreover, the curve of the graph shows that the cytopathogenic rate obtained for this dilution seems to reach a plateau of maximum toxicity (Fig. 3).

### Effect of toxin preincubation with polymyxin B on cytotoxicity

**Figure 4:**
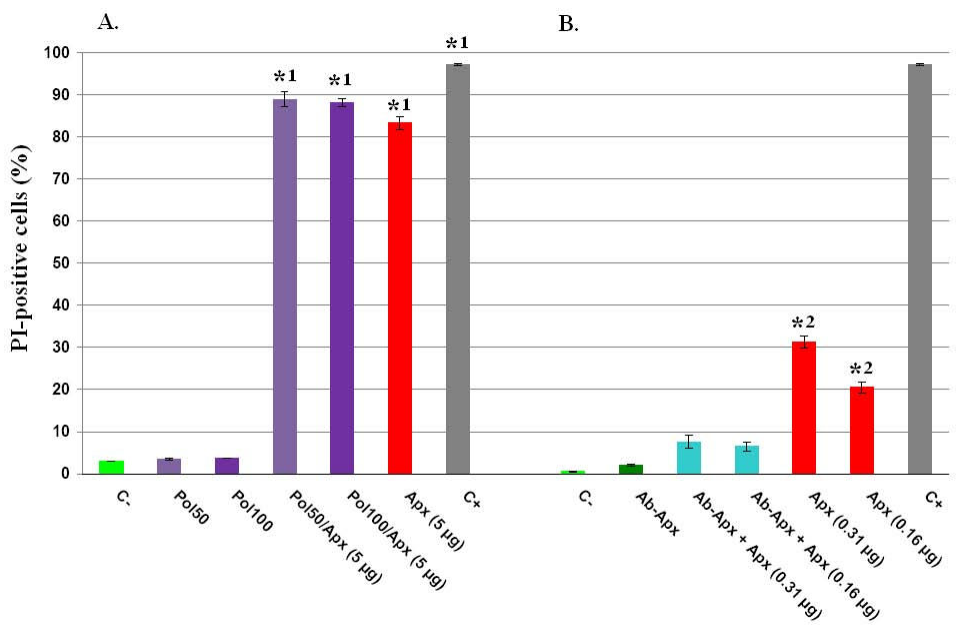
**A. Effect of polymyxin B incorporation on rApxIIIA-containing crude toxin preparation cytotoxicity towards porcine PBMCs**. The fraction of PI-positive cells was measured by flow cytometry after a two-hour exposition (i) to 50 μl RPMI-1640 (C-), (ii) to 50 μg/ml or 100 μg/ml polymyxin B alone (Pol50 and Pol100 respectively), (iii) to 50 μg/ml or 100 μg/ml polymyxin B supplemented with 5 μg rApxIIIA during the second hour (Pol50/Apx and Pol100/Apx), or after a one-hour exposition either (iii) to 5 μg rApxIIIA alone (Apx) or (iv) to paraformaldehyde 10% (C+). Values are means ± SDs from three representative experiments. Asterisks (1): PI-positive cell densities significantly higher than that recorded in C-, Pol50 and Pol100 sets of experiments (*P *< 0.0001). PI-positive cell densities retrieved from Pol50/Apx, Pol100/Apx and rApxIIIA groups were not statistically different from each other (*P *> 0.5). B. Effect of anti-ApxIIIA antibodies incorporation on rApxIIIA-containing crude toxin preparation cytotoxicity towards porcine PBMCs. The fraction of PI-positive cells was measured by flow cytometry after a one-hour exposition (i) to 50 μl RPMI-1640 (C-), (ii) to a monospecific anti-ApxIIIA polyclonal antiserum diluted 1/1,000 (Ab-Apx), (iii) to a mix of rApxIIIA (0.31 or 0.16 μg) and the anti-ApxIIIA polyclonal antiserum diluted 1/1,000 (Ab-Apx + Apx), and (iv) to rApxIIIA alone (Apx) (0.31 or 0.16 μg). Values are means ± SDs from three representative experiments. Asteriks (2): positive cell densities significantly higher than that recorded in Ab-Apx + Apx sets of experiments (*P *< 0.01).

In order to examine whether the LPS content possibly present in the crude toxin preparation contributed to the cytotoxicity detected, the toxin preparation was preincubated during one hour at 37°C with polymyxin B (50 and 100 μg/ml) (Sigma, Belgium) before assaying its cytotoxic activity towards porcine PBMCs. This assay clearly shows that preincubation with polymyxin B does not decrease the cytotoxicity rate and thus, that lipopolysaccharides potentially present in the toxin preparation are not responsible of cell injury induction (Fig. 4A). As negative control, porcine PBMCs were incubated, during the same duration, with the same polymyxin B concentrations without adding the toxin solution.

### Cytotoxicity is due to rApxIIIA

Ultimate assignment of the cytotoxic activity to rApxIIIA was made by neutralizing the crude toxin preparation effect via a preincubation of 30 minutes at 4°C with a rabbit monospecific polyclonal antibody directed against ApxIIIA (1/1,000) friendly provided by Professor J. Frey (Institute of Veterinary Bacteriology, University of Bern, Switzerland) [23]. Incubation of the PBMCs during one hour at 37°C with the preincubated mix (crude toxin preparation/antiserum) resulted in a dramatic abatement in the cytopathogenic activity of two different rApxIIIA amounts (0.16 and 0.31 μg), these latter respectively causing ~30 and ~50% of the maximum cell damage recorded (Fig. 3) when the antiserum was omitted (Fig. 4B). Incubating porcine PBMCs in the same conditions with the monospecific polyclonal antiserum directed against ApxIIIA did not alter the baseline cell death rate (Fig. 4B). These results suggest that the cytotoxic activity associated with our crude toxin preparation is due to rApxIIIA.

### rApxIIIA-induced cytotoxicity is species-specific

**Figure 5:**
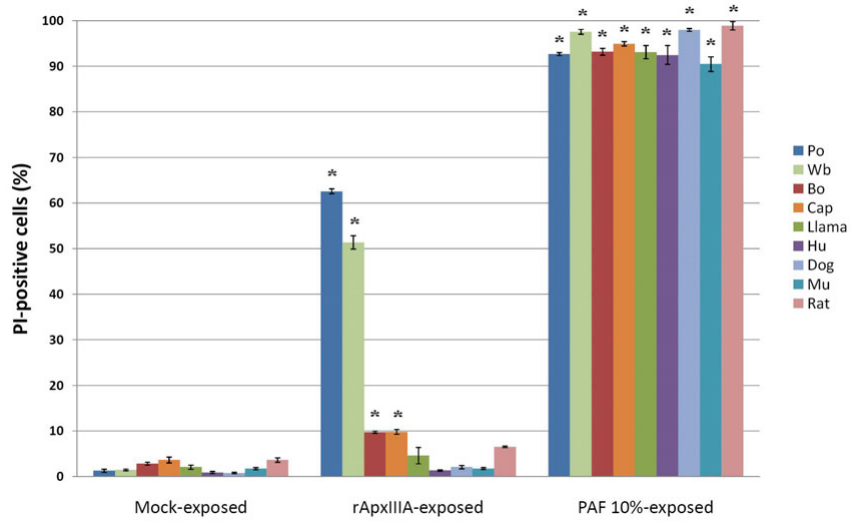
**Cytotoxicity of rApxIIIA-containing crude toxin preparation towards PBMCs from a set of mammalian species**. The fraction of PI-positive cells was measured by flow cytometry after a one-hour exposition to 50 μl RPMI-1640 (mock), 5 μg rApxIIIA or PAF 10%. Values are means ± SDs from three representative experiments. Asterisks: species-specific values significantly different from corresponding mock-exposed value (*P *< 0.05). Po, *Sus scrofa domesticus *Piétrain; Wb (wild boar), *Sus scrofa scrofa*; Bo, *Bos taurus*; Cap, *Capra hircus*; Llama, *Lama pacos*; Hu, *Homo sapiens*; Dog, *Canis familiaris*; Mu, *Mus musculus*; Rat, *Rattus norvegicus*.

When 10^5 ^PBMCs purified from pigs, wild boars, cows, goats, llamas, man, dogs, mice and rats were exposed for one hour to 5 μg rApxIIIA crude toxin, striking differences in susceptibility were enlightened (Fig. 5). Human, llama, dog, mouse and rat PBMCs were totally resistant, whereas bovine and caprine PBMCs showed slight susceptibility (~10% PI-positive cells) compared to pig (~60%) and wild boar (~50%) cells.

### rApxIIIA-induced cytotoxicity is leukocytes-specific

Finally, exposing PK15 porcine epithelial cells to 5 μg rApxIIIA did not result in the induction of cell death, which suggests that cytotoxic activity is restricted to porcine leukocytes (data not shown).

## Conclusion

We have produced a recombinant ApxIIIA toxin in order to evaluate its possible cytotoxic activity towards porcine PBMCs. After one-hour duration incubation at 37°C, PBMCs show a reduction in size and accumulate propidium iodide, both characteristics being compatible with the development of cell death. The cytotoxicity is dose-dependent, develops within minutes, is not susceptible to polymyxin B and is dramatically abated by toxin preincubation with a monospecific polyclonal antiserum directed against ApxIIIA, which suggests that the cytopathogenic activity detected is exercised by ApxIIIA. The cytotoxicity recorded is specifically directed towards porcine and wild boar PBMCs, even if ruminant leukocytes show slight susceptibility too. Overall, we have shown that ApxIIIA shares many functional characteristics with other RTX toxins, i.e. LtxA from *A. actinomycetemcomitans *[24], LktA from *M. haemolytica *[25], HlyA from *E. coli *[26] or CyaA from *B. pertussis *[27], which suggests that it might use the same receptors, the leukocyte *β*_2_-integrins [11-18]. Recombinant expression of porcine CD11a [28] and CD18 [29] in ApxIIIA-resistant cells could answer this question in the future.

## Abbreviations

Apx: *Actinobacillus pleuropneumoniae *RTX toxin; Bo: bovine; BSA: bovine serum albumin; DPBS: Dulbecco's phosphate buffer saline; Hu: human; LB: Luria-Bertani; LPS: lipopolysaccharides; Mu: murine; PBMC: peripheral blood mononucleated cells; PI: propidium iodide; PFT: pore-forming toxin; Po: porcine; RPMI-1640: Roswell Park Memorial Institute-1640; RTX: repeats in toxin; Wb: wild boar.

## Competing interests

The authors declare that they have no competing interests.

## Authors' contributions

PVB carried out the design of the study, experiments and the manuscript drafting. TF and LZ participated in the design of the study. DD participated in the design of the study and coordination, and helped to draft the manuscript. All authors read and approved the final manuscript.
